# Olaparib is effective in combination with, and as maintenance therapy after, first‐line endocrine therapy in prostate cancer cells

**DOI:** 10.1002/1878-0261.12185

**Published:** 2018-03-15

**Authors:** Gertrud E. Feiersinger, Kristina Trattnig, Peter D. Leitner, Fabian Guggenberger, Alexander Oberhuber, Sarah Peer, Martin Hermann, Ira Skvortsova, Jana Vrbkova, Jan Bouchal, Zoran Culig, Frédéric R. Santer

**Affiliations:** ^1^ Division of Experimental Urology Department of Urology Medical University of Innsbruck Austria; ^2^ Department of Anaesthesia and Intensive Care Medical University of Innsbruck Austria; ^3^ Department of Radiotherapy and Radiation Oncology Medical University of Innsbruck Austria; ^4^ Institute of Molecular and Translational Medicine Faculty of Medicine and Dentistry Palacky University Olomouc Czech Republic

**Keywords:** combination therapy, endocrine therapy, maintenance therapy, olaparib, PARP inhibition, prostate cancer

## Abstract

A number of prostate cancer (PCa)‐specific genomic aberrations (denominated BRCAness genes) have been discovered implicating sensitivity to PARP inhibition within the concept of synthetic lethality. Recent clinical studies show favorable results for the PARP inhibitor olaparib used as single agent for treatment of metastatic castration‐resistant PCa. Using 2D and 3D cell culture models mimicking the different treatment and progression stages of PCa, we evaluated a potential use for olaparib in combination with first‐line endocrine treatments, androgen deprivation, and complete androgen blockade, and as a maintenance therapy following on from endocrine therapy. We demonstrate that the LNCaP cell line, possessing multiple aberrations in BRCAness genes, is sensitive to olaparib. Additive effects of olaparib combined with endocrine treatments in LNCaP are noted. In contrast, we find that the TMPRSS2:ERG fusion‐positive cell lines VCaP and DuCaP do not show signs of synthetic lethality, but are sensitive to cytotoxic effects caused by olaparib. In consequence, additive effects of olaparib with endocrine therapy were not observable in these cell lines, showing the need for synthetic lethality in combination treatment regimens. Additionally, we show that PCa cells remain sensitive to olaparib treatment after initial androgen deprivation implicating a possible use of olaparib as maintenance therapy. In sum, our preclinical data recommend olaparib as a synthetic lethal treatment option in combination or sequenced to first‐line endocrine therapy for PCa patients with diagnosed BRCAness.

**Abbreviations**
ADT: androgen deprivation therapy
CAB: complete androgen blockade
NGC: normal growth conditions
RCCS: rotary cell culture system

## 1 Introduction

Current treatment options for metastatic prostate cancer (mPCa) are based on endocrine therapy and taxane‐based chemotherapy. Although second‐generation drugs such as abiraterone acetate (CYP17A1 inhibitor), enzalutamide (androgen receptor (AR) ligand binding domain inhibitor), and cabazitaxel have been introduced in clinical management of PCa, primary or acquired treatment resistances invariably occur (Attard *et al*., 2008; Tran *et al*., 2009). Thus, there is an urgent need to develop novel therapies for mPCa hitting molecular targets different from AR and microtubuli. Such novel treatment options should include (a) single treatment agents, (b) combination treatments with current endocrine and chemotherapy protocols, and (c) maintenance therapies succeeding an initial tumor‐regressive therapy to keep tumor growth and dissemination under control. One compound with the potential to fulfill all three criteria is olaparib (Lynparza™), a first‐in‐class DNA repair inhibitor approved for *BRCA*‐mutated advanced ovarian cancer with prior three or more chemotherapies (Kim *et al*., 2015). Clinical efficacy of olaparib in PCa is currently investigated in several trials with encouraging results as, for example, seen in the TOPARP‐A trial (Mateo *et al*., 2015).

Olaparib inhibits the catalytic activities of key enzymes in single‐strand DNA (ssDNA) break repair, namely poly (ADP‐ribose) polymerases (PARP) 1 and 2 (Menear *et al*., 2008). This can trap PARP on damaged DNA leading to the formation of cytotoxic DNA‐PARP complexes (Murai *et al*., 2012). Initially, PARP inhibitors (PARPi) have been developed in the context of synthetic lethality. Inhibition of ssDNA break repair by PARPi leads to accumulation of chromosomal aberrations during DNA replication and consequently to cell death in cells with defects in homologous recombination (HR) repair mechanisms, termed BRCAness (Lord and Ashworth, 2016). Over the past years, a number of molecular aberrations causing BRCAness have been discovered in PCa and other cancer types, including germline (frequency: 5.3%) or somatic BRCA2 (12.7%), ATM (5.3%), PTEN (40.7%), SPOP (10%), and CHD1 (8%) [frequencies in metastatic castration‐resistant PCa (mCRPCa)] aberrations (Boysen *et al*., 2015; Mendes‐Pereira *et al*., 2009; Pritchard *et al*., 2016; Robinson *et al*., 2015; Shenoy *et al*., 2017). Moreover, gene fusions with the ETS transcription factor family, such as TMPRSS2:ERG (frequency of ~50% in PCa), have been described to drive double‐strand DNA (dsDNA) break formation and thus sensitize cells to PARPi (Brenner *et al*., 2011). Rearrangements with TMPRSS2 put ETS family members such as ERG under transcriptional control of the AR signaling axis. In general, PARP‐1 activity is increased in PCa and is required for AR activity and tumor cell growth (Schiewer *et al*., 2012). Altogether, these molecular aberrations can predict sensitivity to PARPi in PCa patients, and hence, treatment protocols based on the concept of synthetic lethality should encompass selection of the patients based on cancer‐associated BRCAness markers in a personalized medicine approach.

In the present preclinical study, we have addressed the questions whether olaparib, in addition to its usability as a single treatment agent, could be of additive value in a combination treatment with first‐line androgen deprivation (ADT) or complete androgen blockade (CAB) therapy, and whether olaparib could be used as maintenance therapy after initial androgen deprivation.

## 2 Materials and methods

### 2.1 Cell lines and culture

Table S1 gives an overview on the prostate cell lines, culture media, and supplements used in this study. All cell lines were purchased from the American Type Culture Collection (ATCC)—LGC Standards (Wesel, Germany), except BPH1 and DuCaP (gifts from JA Schalken, Nijmegen, the Netherlands), EP156T (Kogan *et al*., 2006), LAPC‐4, and PC3AR (gifts from A Cato, Karlsruhe, Germany). Cell lines were cultured under standard growth conditions with once or twice passaging per week. To mimic ADT and CAB LNCaP, DuCaP and VCaP were cultured in T75 or T175 flasks for ≥ three weeks in +ABL or +BIC conditions, respectively (Table S1), and then seeded into 6‐ or 96‐wells for subsequent experiments. LNCaP‐ABL^Res^ (Culig *et al*., 1999), LNCaP‐BIC^Res^ (Hobisch *et al*., 2006), and DuCaP‐BIC^Res^ were generated by long‐term culture under +ABL or +BIC conditions causing cell cycle arrest/induction of cell death. Upon resuming proliferation, cell lines were considered resistant to +ABL or +BIC conditions and then constantly grown and passaged under +ABL or +BIC conditions, respectively.

### 2.2 Reagents and chemicals

All three PARP inhibitors rucaparib (S1098), veliparib (S1004), and olaparib (S1060) were purchased from SelleckChem (Eubio, Vienna, Austria) and dissolved in DMSO at a stock concentration of 5 mm. R1881 (methyltrienolone, E3164‐000) was purchased from Serobac (Vienna, Austria) and dissolved in ethanol at 10–100 nm stock concentrations. Bicalutamide (B9061) was ordered from Sigma‐Aldrich (Vienna, Austria) and dissolved in DMSO at 1 mm stock concentration.

### 2.3 Rotary cell culture assays

The rotary cell culture system RCCS‐4D (Synthecon/Cellon, Bascharage, Luxembourg) with 10‐mL disposable vessels rotated at 45 RPM was used to grow cells in three dimensions. Vessels were inoculated with 3 × 10^6^ cells in suspension in normal growth medium, and rotary cell culture was started. After 24 h, small organoids were observable. Conditioned medium was removed from the vessels at the timepoints two, six, and nine days without disturbing the organoids and replaced with NGC or +ABL medium containing 5 μm olaparib or DMSO. PSA was determined in the conditioned medium as described (Data S1) and normalized on incubation time. On day nine, organoids were removed from the vessels and embedded in coagulated citrate‐plasma (450 μL citrate‐plasma, 45 μL thrombin 120 NIH‐U·mL^−1^, 11.3 μL 1 m calcium chloride). Organoids were fixed in buffered 4% formaldehyde for 2 h at room temperature, dehydrated in a Tissue‐Tek VIP (Sakura, Vienna, Austria), stained with eosin to visualize organoids, and embedded in paraffin. Two‐micrometer sections were prepared using a microtome, and consecutive sections were stained for MKI67 (anti‐Ki‐67, MIB‐1; 1 : 400, M7240, Dako, Agilent, Vienna, Austria) and cleaved CASP3 (anti‐cleaved caspase 3 (Asp157), 1 : 400, #9661, Cell Signaling, New England Biolabs, Frankfurt a. M., Germany) using a Discovery‐XT staining device (Ventana, Tucson, AZ, USA) with CC1 antigen retrieval conditions. Sections were counterstained with hematoxylin, and images were taken on an Axio Imager Z2 microscope (Carl Zeiss, Vienna, Austria) equipped with a TissueFAXS automated recording system (TissueGnostics, Vienna, Austria) at 20× magnification. Overview images were generated by stitching neighboring 20x images.

For tumor‐initiation experiments in the RCCS‐4D, vessels were inoculated with the indicated number of LNCaP + ABL cells in NGC and treated with DMSO or 5 μm olaparib. After ten days, incubation at 45 RPM PSA in the supernatant was measured and tumor organoids were collected using a 70‐μm cell strainer (BD, Schwechat, Austria). Organoids were documented with a stereomicroscope (Nikon, Vienna, Austria).

### 2.4 Statistical analysis

prism 5 (GraphPad Software, La Jolla, CA, USA) and STATISTICA (StatSoft, Hamburg, Germany) were used to perform Student′s *t*‐test, Mann–Whitney test, and Friedman test, as well as one‐way and two‐way ANOVA followed by Bonferroni's multiple comparison test. Figure legends inform on the statistical test used. Data are presented as mean of multiple experiments (*n* ≥ 3) ± standard error of the mean (SEM) to estimate how variable the means in multiple repeated experiments were, unless indicated otherwise. Statistically significant differences are encoded in the figures as follows: ns, not significant; *P < 0.05; **P < 0.01; ***P < 0.001.

Data S1 are available online.

## 3 Results

### 3.1 Screening of a panel of prostate (cancer) cell lines for PARPi sensitivity

**Figure 1:**
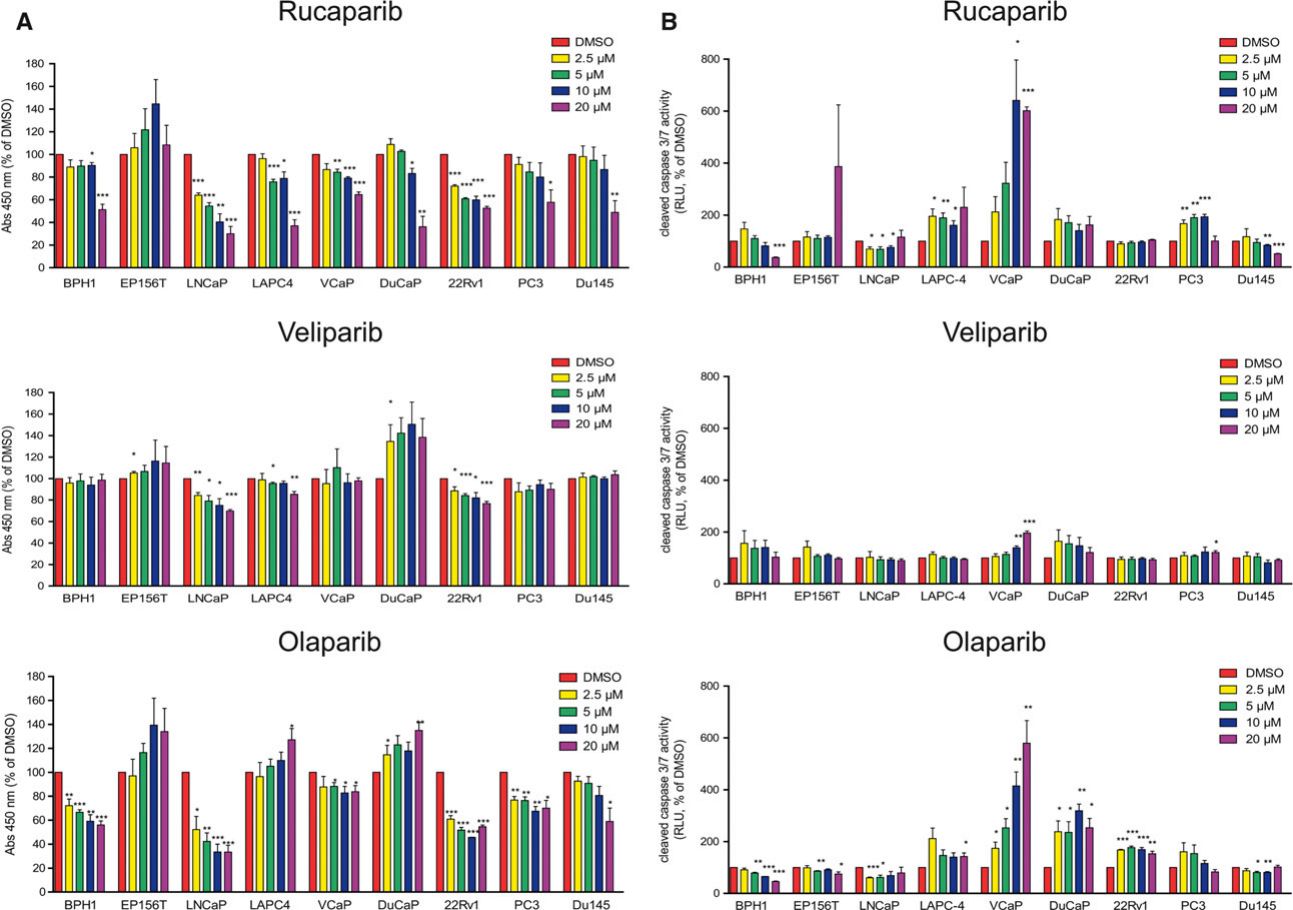
Efficacy of rucaparib, veliparib, and olaparib on various prostate immortalized benign and cancer cell lines. A panel of immortalized benign (BPH1, EP156T) and malignant (LNCaP, LAPC4, VCaP, DuCaP, 22Rv1, PC3, and Du145) prostate cell lines were treated for 96 h with increasing concentrations (2.5–20 μm) of rucaparib, veliparib, or olaparib, or the same volume of vehicle (DMSO), as indicated. (A) Viability was assessed by MTT assay. (B) Induction of apoptosis was assessed by cleaved CASP 3/7 activity assay. (A, B) Statistically significant differences were calculated by Student's *t*‐test. *P < 0.05; **P < 0.01; ***P < 0.001.

In order to identify prostate cancer cell lines sensitive to PARPi treatment, we performed a screening with a panel of available cell lines cultured under NGC. Immortalized benign cell lines BPH1 and EP156T, as well as AR‐positive LNCaP, LAPC4, VCaP, DuCaP, and 22Rv1 and AR‐negative PC3 and Du145 PCa cell lines, were exposed for 96 h with increasing concentrations (0–20 μm) of three commercially available PARPi: rucaparib, veliparib, and olaparib. Sensitivity to PARPi was assessed by MTT viability (Fig. 1A) and for apoptosis induction using cleaved CASP3/7 activity assays (Fig. 1B). Comparison of the three PARPi showed a more potent activity of rucaparib and olaparib relative to veliparib and on a higher number of cell lines in both viability and apoptosis assays. Statistically significant differences with all three PARPi were found in LNCaP and 22Rv1 in viability and in VCaP in apoptosis assays. Next, we screened the mutation and expression profile of the COSMIC cell line database for a list of BRCAness genes known or susceptible to confer sensitivity to PARPi and/or to be involved in homologous recombination (Forbes *et al*., 2015). Analysis of the mutational database identified that LNCaP, 22Rv1, and Du145 harbor >25 mutated genes implicated in sensitivity to PARPi including mutations in *BRCA1/2* genes (Table 1A, Table S2). Detailed analysis of the pathogenic status of *BRCA1/2* genes revealed that LNCaP and 22Rv1 harbor *BRCA2* mutations with a high probability to confer PARPi sensitivity, while mutations in *BRCA1/2* in Du145 have a low probability (Table S2, pathogenicity of BRCA mutations). Evaluation of the expression database encompassing copy number variations showed differential expression of a number of BRCAness genes among all cell lines (Table 1B, Table S3). Based on this and published data, we decided to further analyze PARPi sensitivity of the androgen withdrawal‐sensitive cell lines LNCaP, VCaP, and DuCaP (the latter two being TMPRSS2:ERG2 fusion‐positive). Among PARPi tested, olaparib showed the highest activity on these cell lines (Fig. 1) and was selected for further investigations.

**Table 1:** Mutational profile (A) and expression levels (B) of selected genes involved in HR and BRCAness genes in PCa cell lines. The mutations and CNV & Expression COSMIC Cell lines project's databases were screened for known mutations and for expression levels (*Z*‐scores >2 or <−2 are shown), respectively, using a list of HR and BRCAness genes on the indicated cell line. BRCA2 mutations (indicated with an asterisk) in LNCaP and 22Rv1 have a high probability for pathogenicity. For details, see Tables S2 and S3

BPH1		LNCaP		VCaP		22Rv1		PC3		Du145	
**A**											
None		ABL1		SSRP1		AR		GTF2H3		BRCA1	
		AR				ATAD5		SMARCAL1		BRCA2	
		ATM				ATM				BRIP1	
		ATR				BARD1				CDK7	
		BARD1				BRCA2*				DDB1	
		BRCA2*				CDK7				FANCB	
		BRIP1				CHD1				FANCI	
		CHD1				DNMT3A				FLI1	
		CHEK2				EME1				IPMK	
		DDB1				FANCM				LIG4	
		DNMT3A				FLI1				MAPK12	
		ERCC1				HELLS				MMS22L	
		ESCO1				INO80D				PAPD7	
		ETV1				KAT5				POLQ	
		FANCA				LIG4				RAD50	
		FANCE				MCM3				RBBP8	
		G2E3				MUM1				REV3L	
		GTF2H3				NBN				SLX4	
		MMS22L				PALB2				SMARCA2	
		MUM1				RAD54L				SSRP1	
		PAPD7				SHPRH				STK36	
		PLK3				SLX4				TP53BP1	
		POLB				SMG1				UBE2N	
		POLH				STK36				USP1	
		PTEN				TP53BP1				XRCC2	
		RAD51B				USP1					
		RAD54L				USP7					
		RECQL4				WRN					
		SMARCA5				XRCC2					
		SPO11									
		SSRP1									
		TOP2B									
		TP53BP1									
		UBA1									
		XRCC2									
**B**											
CHEK2	−2.283	AR	3.51	AR	5.181	AR	4.466	BRCA1	−2.513	FANCG	3.231
ETV1	−2.326	DDB1	−2.518	ATR	−2.619	INO80D	2.102	CDK5	2.591	G2E3	2.971
FAM175A	−2.307	DNASE1L2	2.124	ERG	3.802	MSH3	−4.015	MUM1	−3.271	MUS81	2.05
GADD45A	2.262	ETV1	2.454	KAT5	3.541	RNF168	−2.512	PNKP	2.18	XRCC3	2.112
LIG3	−2.599	PRMT6	3.644	LIG3	3.149	SHPRH	2.36	PTEN	−3.164		
UNG	2.724	TMPRSS2	3.981	MUS81	2.202			RAD50	−2.662		
		XRCC3	2.428	PALB2	−3.094			RECQL4	2.449		
				POLK	−2.939			SMG1	−2.483		
				RAD23B	3.623			SPO11	−2.246		
				SMG1	−2.385						
				TMPRSS2	2.76						
				UBE2A	−5.143						
				USP10	−5.321						

### 3.2 Additive effects of olaparib combined to endocrine therapy

**Figure 2:**
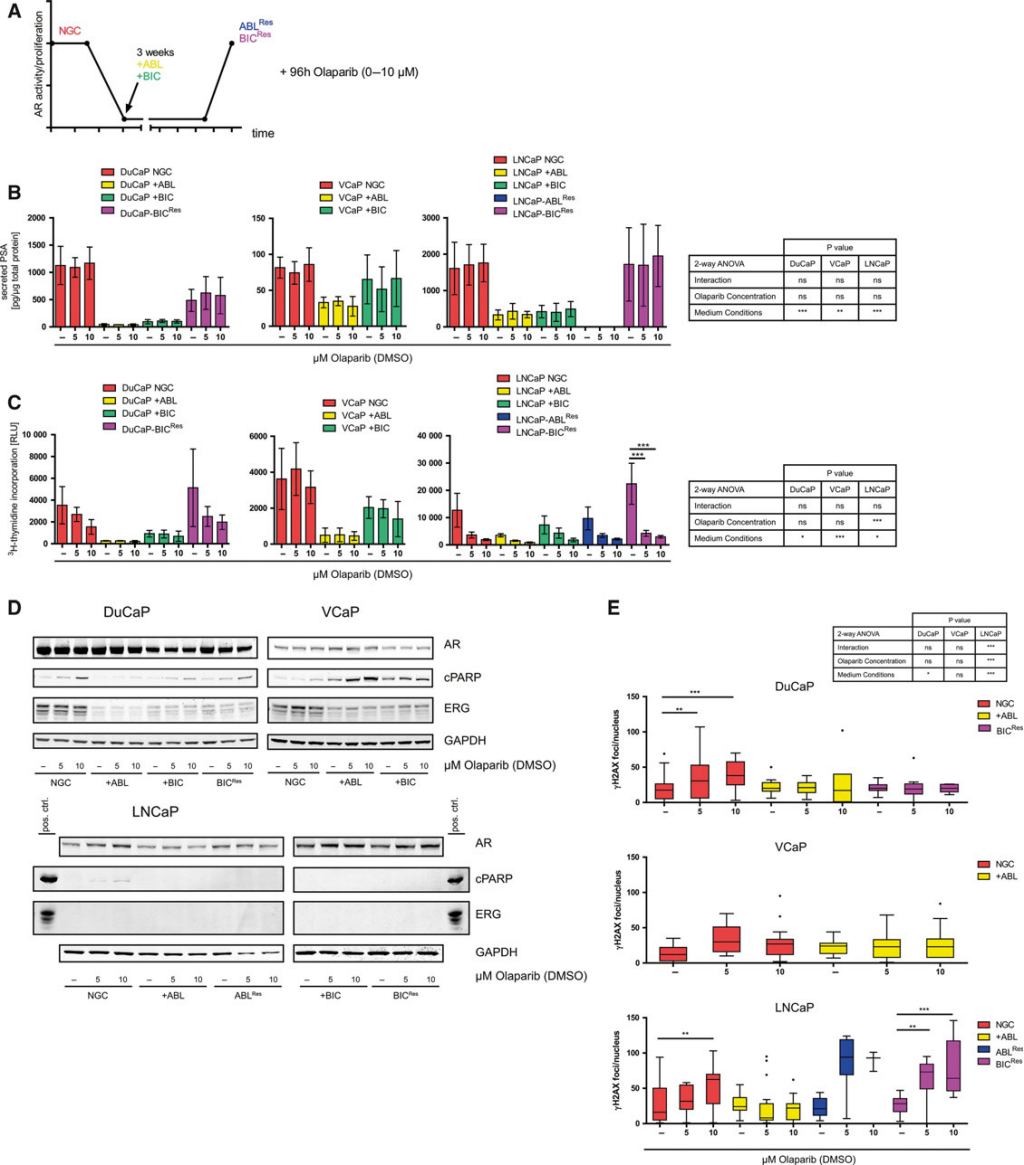
Efficacy of short‐term treatment with olaparib on androgen‐sensitive prostate cancer cell lines mimicking different therapeutic options and disease progression stages. (A) Scheme depicting the various treatment conditions and progression stages and their effects on AR activity and cellular proliferation. Experiments in this figure were conducted with DuCaP, VCaP, and LNCaP that were treated with olaparib for 96 h. (B) Secreted PSA was measured in the supernatant and normalized to total protein content of producing cells, as indicated. (C) Proliferation was measured by ^3^H‐thymidine incorporation assay. (D) Immunoblotting was performed to determine expression levels of the apoptosis marker cPARP, as well as AR and ERG. GAPDH served as equal loading control. A representative immunoblot of at least three independent experiments is shown. Positive controls for cPARP and ERG have been loaded in adjacent gel lanes in LNCaP immunoblots. (E) Immunofluorescence assay for γH2AX was performed to assess the induction of dsDNA breaks. The number of γH2AX foci per nucleus was assessed, and absolute values are depicted as box‐and‐whisker plots. (B, C, E) Statistically significant differences were calculated by two‐way ANOVA test (table) with Bonferroni post‐test (stars in graph). (A–E) NGC, normal growth conditions; +ABL, androgen deprivation mimicking ADT; +BIC, bicalutamide treated mimicking CAB; ABL^R^ ^es^, resistant to androgen ablation; BIC^R^ ^es^, resistant to bicalutamide treatment; RLU, relative light units. *P < 0.05; **P < 0.01; ***P < 0.001.

In theory, PARPi could be clinically used in different progression stages with different treatment regimens of PCa, that is, prior (treatment‐naïve), during (combination therapy), and posterior (CRPC) endocrine therapy. These stages of endocrine therapy were mimicked by manipulating androgen concentrations in the culture medium (for culture conditions, see Table S1). To this end, the medium was switched from NGC to +ABL (mimicking ADT) or +BIC (mimicking CAB) (Fig. 2A). To mimic CRPC, cells were cultured under long‐term (up to nine months) +ABL or +BIC conditions. Upon resuming proliferation, cells were considered castration‐resistant and named −ABL^Res^ or −BIC^Res^. Unfortunately, we were unable to generate DuCaP‐ABL^Res^ and VCaP‐ABL^Res^/‐BIC^Res^ cell lines. The effects of olaparib (0–10 μm, 96 h incubation) were assessed under these conditions. While +ABL and +BIC had strong effects on PSA secretion in DuCaP and LNCaP, VCaP cells were less affected by these conditions (Fig. 2B). DuCaP‐BIC^Res^ and LNCaP‐BIC^Res^ resumed PSA secretion, which was not seen in LNCaP‐ABL^Res^. Importantly, olaparib did not have any effect on PSA secretion under either stage. Next, the effect of olaparib on cellular proliferation under the different stages was elaborated (Fig. 2C). Culture under +ABL and +BIC conditions resulted in markedly decreased proliferation rates, while proliferation rate was resumed or increased (compared to NGC) in ABL^Res^ and BIC^Res^. Olaparib treatment decreased proliferation of LNCaP under either condition while proliferation of VCaP was not influenced by olaparib treatment. Furthermore, expression levels of AR and ERG, as well as the apoptosis marker cPARP, were assessed by immunoblotting (Fig. 2D). Culture of DuCaP and VCaP under +ABL and +BIC conditions resulted in a stark decrease of the androgen‐regulated TMPRSS2:ERG fusion protein. Interestingly, re‐expression of ERG in DuCaP‐BIC^Res^ was not observed. Apoptosis induction by olaparib under NGC was seen in DuCaP and, less pronounced, in VCaP, while almost absent in LNCaP. Contrasting results of olaparib on cPARP levels were also observed under +ABL and +BIC conditions: Apoptosis induction in DuCaP was markedly decreased compared to NGC, while VCAP showed increased levels. No apoptosis induction was observed in LNCAP. In resistant stages, a mild apoptosis induction by olaparib was observed in DuCaP‐BIC^Res^. Finally, we evaluated the induction of dsDNA breaks by γH2AX immunofluorescence (Figs 2E and [Link], [Link], [Link]). Significant increases in γH2AX foci/nucleus were observed in DuCaP and LNCaP in NGC after olaparib treatment, while +ABL precluded induction of dsDNA breaks by olaparib in all cell lines. LNCaP‐ABL^Res^ and ‐BIC^Res^ showed increased γH2AX levels after olaparib treatment, which was not observed in DuCaP‐BIC^Res^. Importantly, VCaP showed only a mild, nonsignificant induction of γH2AX foci/cell by olaparib under NGC, which was not seen under +ABL conditions. We therefore concluded that induction of apoptosis by olaparib in VCaP+ABL (Fig. 2D) is likely due to cytotoxic effects and not due to generation of dsDNA damage.

### 3.3 Failure of increased ERG expression to sensitize for olaparib treatment

**Figure 3:**
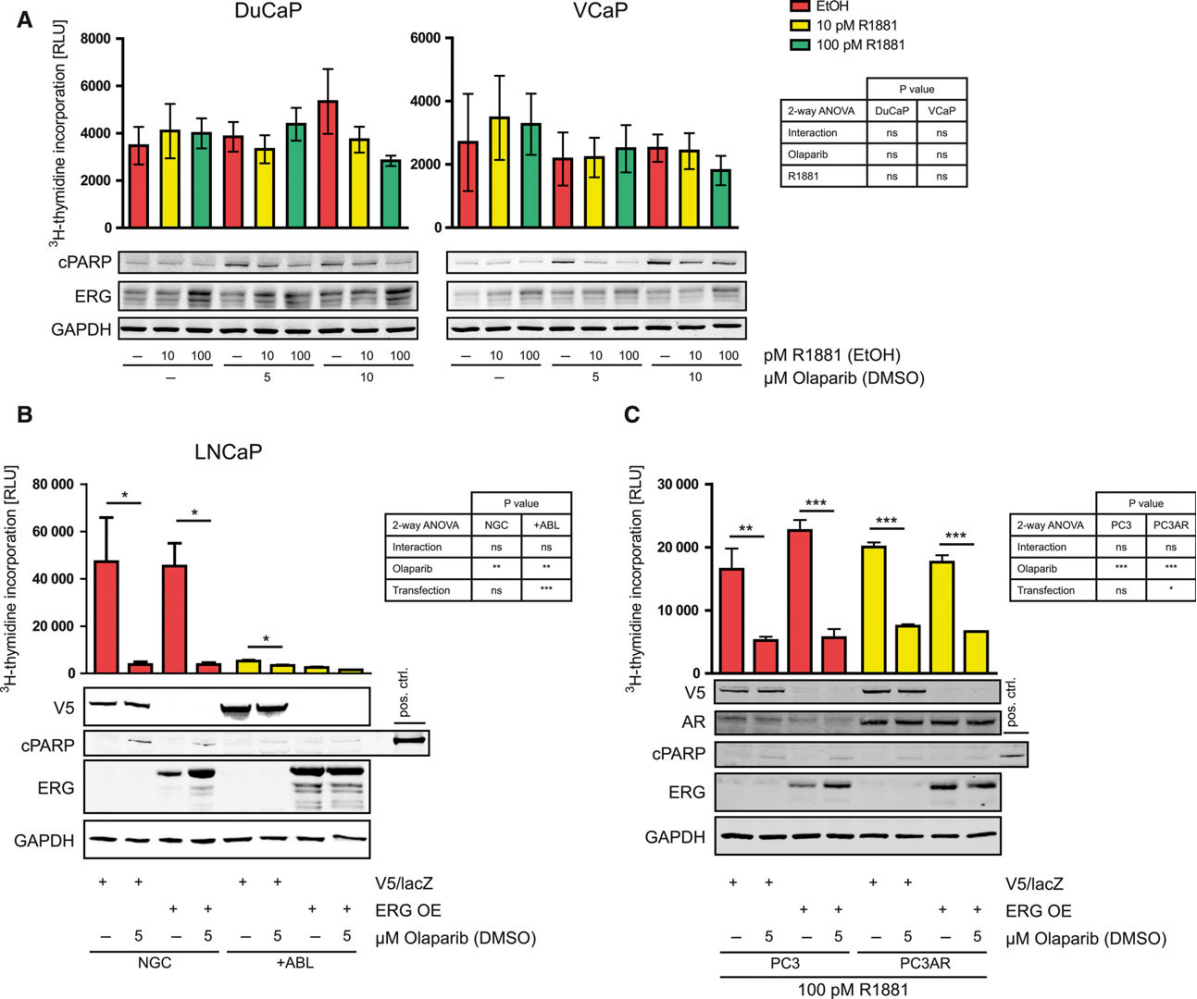
Influence of ERG expression on the sensitivity to olaparib short‐term treatment. (A) DuCaP and VCaP were treated with 10 or 100 pm R1881 or vehicle (EtOH), and 5 or 10 μm olaparib or vehicle (DMSO) for 96 h, as indicated. (B,C) Fusion‐negative LNCaP, PC3, and PC3AR cells were stably transfected with lentiviral constructs to overexpress β‐galactosidase fused to V5 (V5/lacZ, control) or truncated ERG, as found in fusion‐positive cells. LNCaP‐V5/lacZ and –ERG were then treated with 5 μm olaparib or vehicle (DMSO) for 96 h, as indicated. PC3 and PC3AR cells were treated with 100 pm R1881. (A–C) Immunoblotting was performed to determine expression levels of the apoptosis marker cPARP, as well as AR, ERG, or V5/lacZ. GAPDH served as equal loading control. A representative immunoblot of three independent experiments is shown. In parallel, proliferation under those conditions was determined by ^3^H‐thymidine incorporation assay. (A) No statistically significant differences by two‐way ANOVA test were found. (B, C) Two‐way ANOVA (table) with Bonferroni post‐test (stars in graph) was performed separately for NGC and +ABL conditions (B) and for PC3 and PC3AR (C). RLU, relative light units. *P < 0.05; **P < 0.01; ***P < 0.001.

TMPRSS2:ERG fusion could be an important biomarker for PARPi sensitivity due to its high frequency among patients. However, our results showed that ERG expression is not necessary a prerequisite for PARPi sensitivity, as seen, in particular, in VCaP +ABL and +BIC cells (Fig. 2D). To elaborate in more detail the effects of ERG expression on PARPi sensitivity, we treated DuCaP and VCaP with 10 or 100 pm of the synthetic androgen R1881 (Fig. 3A). Although these castrate levels of R1881 were sufficient to increase dose‐dependently ERG expression levels, only minor changes in proliferation indexes could be measured. However, despite increased ERG expression levels, cPARP levels were decreased in samples cotreated with R1881 and olaparib compared to olaparib‐only treated. In addition, stable overexpression of ERG did not increase sensitivity of LNCaP cells to olaparib treatment in either NGC or +ABL conditions, as measured by proliferation and cPARP apoptosis immunoblots (Fig. 3B). To address the effect of ERG expression in the absence of AR expression, PC3 cells were assessed for olaparib sensitivity. PC3 cells stably transfected with a control construct (V5/lacZ) showed decreased proliferation and a very minor induction of apoptosis upon olaparib treatment, which was not affected by AR expression in PC3 stably transfected with AR (PC3AR) (Fig. 3C). Furthermore, ERG overexpression could not increase sensitivity of PC3 to olaparib in neither absence nor presence of R1881‐activated AR.

### 3.4 Increased induction of apoptosis after long‐term incubations with olaparib in LNCaP, but not in TMPRSS2:ERG‐positive cell lines

**Figure 4:**
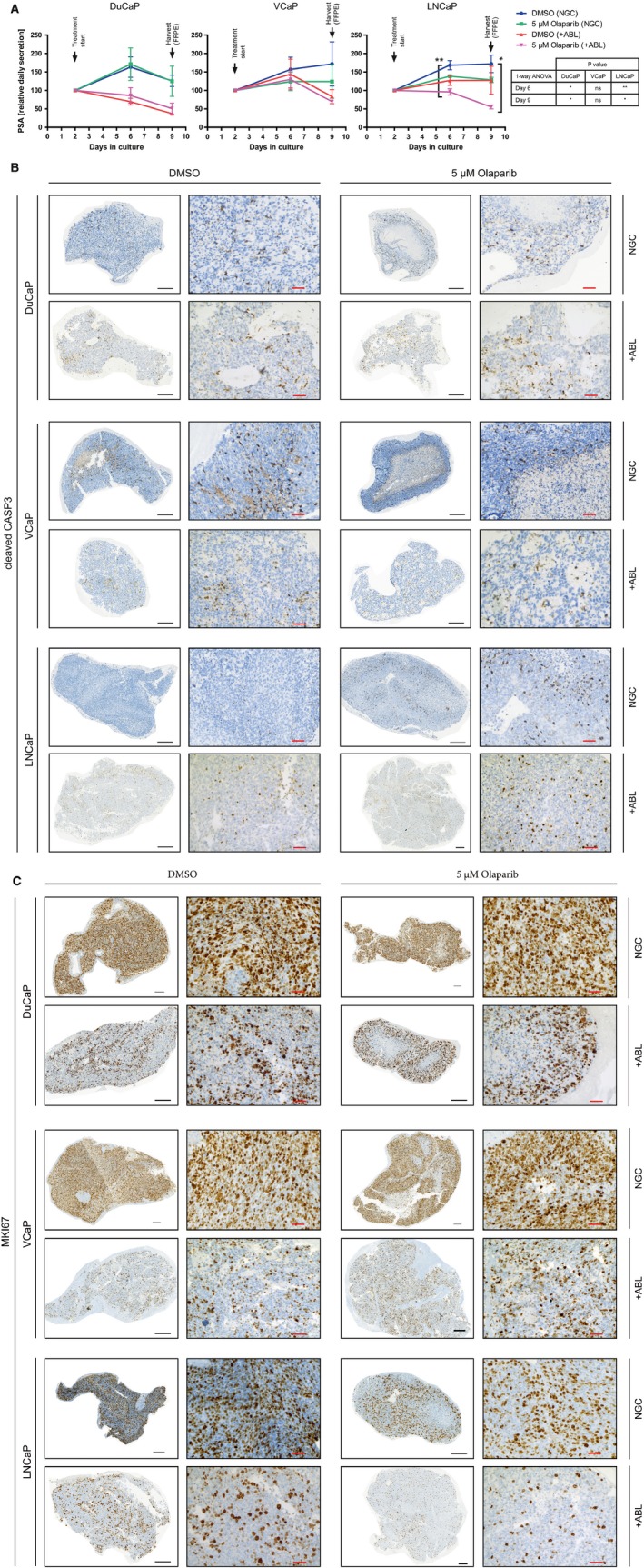
Efficacy of olaparib long‐term treatment on organoids grown in a rotary culture system. Rotary disks were inoculated with dispersed DuCaP, VCaP, and LNCaP cells and incubated at 45 RPM. Organoids formed after 2 days and treatment for 7 days with 5 μm olaparib or vehicle (DMSO) was started, as indicated. Medium was replaced at days 2 and 6. (A) Secreted PSA was measured in the conditioned medium on days 2, 6, and 9, and values are depicted relative to day 2 as secreted PSA per day. One‐way ANOVA (table) with Bonferroni post‐test (stars in graph) was performed to assess statistically significant differences between treatments. (B, C) Nine days after inoculation, organoids were harvested and formalin‐fixed paraffin‐embedded (FFPE) sections were stained for cleaved CASP3 (B) and MKI76 (C). Samples were counterstained with hematoxylin. Representative overview (left) and detail (right) views are shown for each treatment, as indicated. Black scale bar, 200 μm, red scale bar, 50 μm. (A–C) NGC, normal growth conditions; +ABL, androgen deprivation mimicking ADT. *P < 0.05; **P < 0.01.

In order to determine sensitivity to PARPi with longer incubation times and under clinically more relevant, three‐dimensional conditions we made use of a rotary cell culture system (RCCS). Rotating vessels were inoculated with detached DuCaP, VCaP, and LNCaP cells. Growing cellular aggregates were observable after a few hours resulting in up to ten organoids/disk of up to three millimeter in diameter after nine days. Two days after inoculation, treatments with olaparib under NGC or +ABL conditions were started. PSA in the supernatant was measured 2, 6, and 9 days after inoculation (Fig. 4A). PSA secretion of VCaP organoids was at the lower detection limit of the assay and ranged between 0.4978 ± 0.292 ng/(mL × day) [DuCaP: 22.75 ± 16.37 ng/(mL × day) and LNCaP: 43.48 ± 25.08 ng/(mL × day)]. PSA secretion dropped under +ABL conditions compared to NGC in DuCaP and LNCaP organoids. In DuCaP organoids, olaparib treatment did not have any effect on PSA secretion under either condition. In contrast, olaparib treatment of LNCaP organoids resulted in decreased PSA secretion under NGC and an additive effect of olaparib and +ABL was observable. At day nine after inoculation, organoids were harvested and FFPE sections were stained for cleaved CASP3 (Fig. 4B) and MKI67 (Fig. 4C) as markers for apoptosis and proliferation, respectively. Counterstaining with hematoxylin revealed necrotic areas in the center of organoids likely resulting from a lack of supply with dioxygen and nutrients. DuCaP and VCaP organoids demonstrated a higher degree of cleaved CASP3 staining compared to LNCaP under NGC. When grown under +ABL conditions, the number of apoptotic cells did not markedly change in DuCaP and VCaP organoids, but was increased in LNCaP organoids. Olaparib treatment did not affect apoptosis induction in DuCaP and VCaP organoids under either NGC or +ABL. In contrast, increased levels of cleaved CASP3 staining were observed in LNCaP organoids cultured in NGC after olaparib treatment, which increased further when olaparib was combined to +ABL conditions. Stainings with MKI67 revealed a high proliferative index of DuCaP and VCaP organoids, while a number of cells in LNCaP organoids did not actively cycle. When grown under +ABL conditions, proliferation drastically decreased in all three cell line organoids. Treatment with olaparib was able to decrease MKI67 staining in LNCaP organoids under NGC, but unable in DuCaP and VCaP organoids. Again, a combined effect of +ABL and olaparib treatment in LNCaP organoids was observable, but absent in DuCaP and VCaP organoids.

### 3.5 Conserved olaparib efficacy after initial ADT

**Figure 5:**
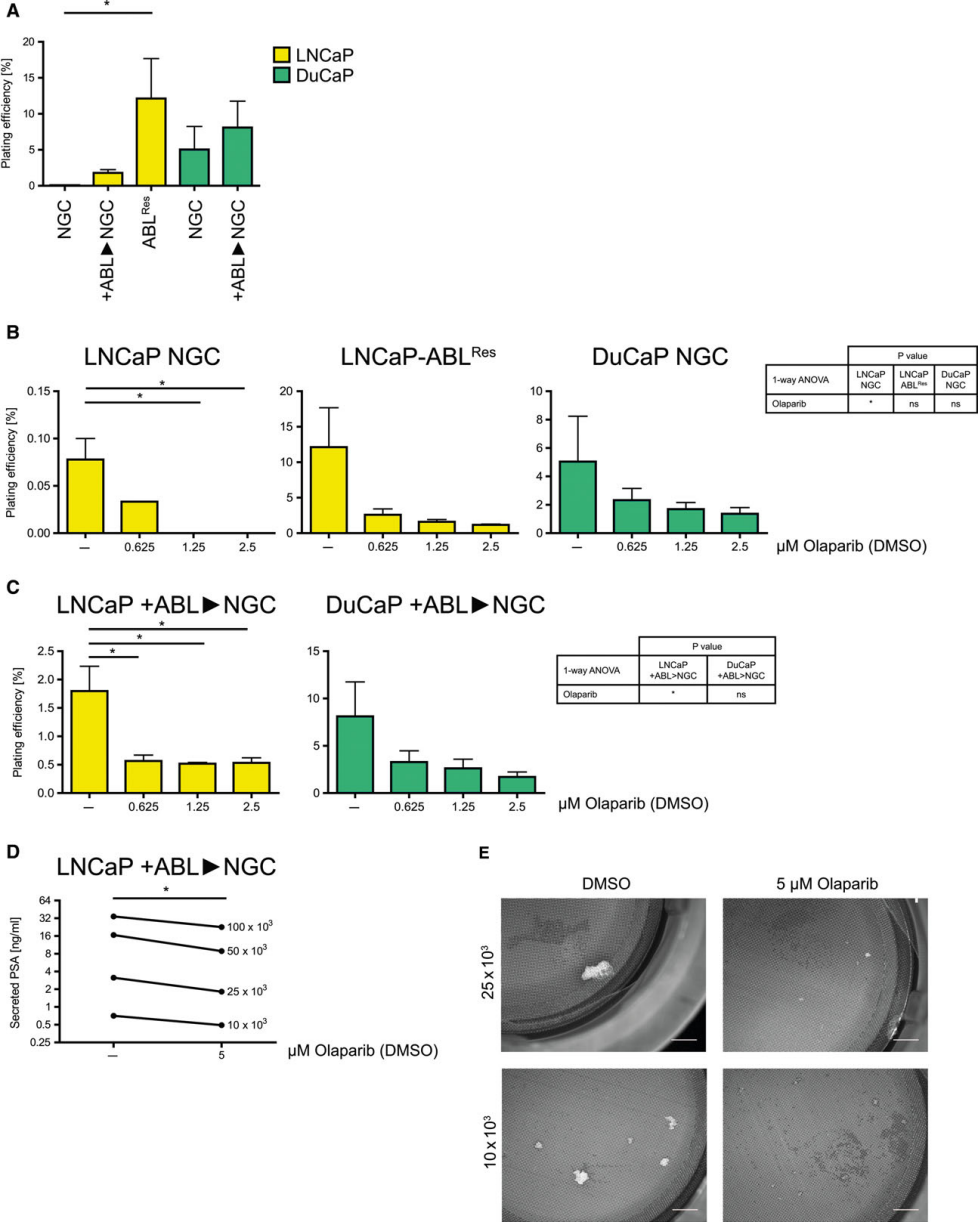
Efficacy of olaparib after initial androgen deprivation. Clonogenic assays were performed with LNCaP, LNCaP‐ABL^R^ ^es^, and DuCaP. In addition, LNCaP and DuCaP were grown for 3 weeks under +ABL conditions and then seeded for clonogenic assay under NGC (indicated as +ABL>NGC). (A) Plating efficiency (ratio of number of colonies per number of seeded cells) is depicted in absolute values. Statistically significant differences were calculated by Mann–Whitney test. (B, C) Clonogenic assays were performed with increasing concentrations of olaparib (0–2.5 μm), as indicated. Plating efficiencies relative to vehicle (DMSO) are indicated, and statistically significant differences were calculated by one‐way ANOVA (table) and Bonferroni post‐test (stars in graphs). (D, E) Tumor‐initiation experiments were performed with 1 × 10^4^–10^5^ LNCaP grown for 3 weeks under +ABL conditions followed by incubation in the RCCS with DMSO or 5 μm olaparib. (D) PSA was measured in the supernatant. Single values resulting from inoculations with different cell numbers are depicted, and statistically significant differences were calculated with Friedman test. (E) Tumor organoids were collected with a cell strainer. Scale bar, 2 mm. (A–D) NGC, normal growth conditions; +ABL, androgen deprivation mimicking ADT. *P < 0.05.

Finally, clonogenic assays with olaparib were performed as an indicator for tumor‐initiation potential. LNCaP NGC, LNCaP‐ABL^Res^, and DuCaP NGC were able to form colonies, while LNCaP–BIC^Res^, DuCaP‐BIC^Res^, and VCaP did not yield clonogenic growth. LNCaP‐ABL^Res^ had a significantly increased plating efficiency compared to LNCaP NGC (Fig. 5A). LNCaP NGC and LNCaP‐ABL^Res^ were sensitive to low concentrations of olaparib (0.625–2.5 μm), and 1.25 μm was sufficient to completely inhibit clonogenicity of LNCaP NGC (Fig. 5B). Plating efficiency of DuCaP NGC was slightly affected by up to 2.5 μm olaparib. To assess whether olaparib might be used as a maintenance therapy replacing ADT after initial tumor regression, we pretreated LNCaP and DuCaP for three weeks under +ABL conditions followed by clonogenic assays under NGC (Fig. 5C). This +ABL selection step led to increased plating efficiency compared to NGC (Fig. 5A). Importantly, olaparib was able to decrease clonogenic growth in LNCaP under these conditions and also in DuCaP albeit statistically nonsignificant. Finally, we made use again of the RCCS to monitor tumor initiation of +ABL‐pretreated LNCaP under NGC conditions. A minimum number of 10x10³ +ABL‐pretreated LNCaP were necessary to yield organoid growth. Determination of secreted PSA (Fig. 5D) and of organoid size (Fig. 5E) showed that olaparib treatment could inhibit organoid growth after initial +ABL conditions.

## 4 Discussion

### 4.1 Rationales for synthetic lethality by PARP inhibition in PCa

To date, few clinical studies assessing PARP inhibitors in m(CR)PCa are available (Fong *et al*., 2009; Kaufman *et al*., 2015; Mateo *et al*., 2015; Sandhu *et al*., 2013). Favorable results have been noted, in particular, in patients with defects in DNA repair genes denominated BRCAness genes. In fact, predicting sensitivity to PARPi for personalized cancer therapy is difficult and encompasses routine screening for germline pathogenic mutations in *BRCA1/2* genes only. However, molecular findings and retrospective genomic analysis of clinical data show that synthetic lethality with PARPi is not restricted to mutant *BRCA1/2* but can also involve other genes. We found that TP53 wild‐type LNCaP cells show an initial proliferative arrest after four‐day olaparib treatment (Fig. 2) and an apoptotic response after seven days (Fig. 4). This shows the necessity for prolonged incubation times to assess synthetic lethality based on generation of DNA damage by PARPi. LNCaP harbor not only a BRCA2 mutation with a high probability of pathogenicity, but also mutations in HR genes RAD51B, RAD54L, ATM, and FANCA (Table 1) showing that multiple genetic aberrations in HR genes could contribute to PARPi sensitivity. Furthermore, LNCaP cells express the fusion TMPRSS2:ETV1, encode mutated CHD1, and are negative for PTEN protein, all of which have been shown to sensitize to PARP inhibition (Brenner *et al*., 2011; Kari *et al*., 2016; Mendes‐Pereira *et al*., 2009; Sharrard and Maitland, 2000). We therefore conclude that the exact mechanism of PARPi sensitization in LNCaP cells is unclear and may likely include more than one single mechanism. Thus, the LNCaP example nicely illustrates the difficulty in stratifying cancer patients that may benefit from PARPi therapy and the need to further investigate synthetic lethality mechanisms with PARPi.

In this work, we focused on the role of TMPRSS2:ERG in mediating synthetic lethality (Brenner *et al*., 2011). The initial hypothesis was to investigate whether the downregulation of ERG, by suppressing AR activity at the TMPRSS2 promoter, is sufficient to abolish PARPi sensitivity of VCaP and DuCaP cell lines. This may have answered the question whether endocrine therapy in fusion‐positive patients restricts sensitivity to olaparib treatment, thus not supporting such a combination therapy. Instead, we found that the number of γH2AX foci/cell is only moderately increased after olaparib treatment in fusion‐positive VCaP compared to LNCaP under NGC (Fig. 2E). After androgen deprivation, an increase in γH2AX foci/cell was not noted in VCaP (Fig. 2E), while markedly increased apoptosis was observable (Fig. 2D). Detailed analysis by ERG expression level manipulation did also not indicate a contribution of ERG to olaparib sensitivity (Fig. 3), and after longer incubation times, olaparib was not able to induce apoptosis in VCaP and DuCaP cells (Fig. 4). In contrast to the study by Brenner *et al*. (2011), we were not able to document decreased proliferation rates when PC3 cells were manipulated to overexpress ERG, nor could we find a sensitization after ERG overexpression to olaparib in this cell line (Fig. 3C). Thus, we speculate that the pro‐apoptotic effects observed in VCaP and DuCaP under NGC (Figs 1, 2D) after four days olaparib treatment are due to cytotoxic effects and possibly a result of olaparib trapping PARP to sites of DNA damage resulting in stalled replication forks during DNA replication (Murai *et al*., 2012). PARP trapping may also contribute to the effects observed in LNCaP and PC3 cells. A higher cytotoxicity of olaparib in VCaP and DuCaP may also be a result of a higher basal apoptotic index as compared to LNCaP (Fig. 4B), which could be caused by genomic instability due to ERG overexpression (Brenner *et al*., 2011) and/or by a gain‐of‐function mutation in TP53 (p.R248W) (van Bokhoven *et al*., 2003; Song *et al*., 2007). In sum, we raise concerns that ERG overexpression in combination with PARPi may not induce synthetic lethality and thus may not serve as biomarker to stratify patients for PARPi therapy. This is underlined by clinical studies (NCT01576172 and NCT00749502) which found no impact of the ETS status on the progression‐free survival (PFS) of patients treated with a combination of abiraterone and veliparib (Hussain *et al*., 2017) or on clinical benefit with the PARPi niraparib administered as single treatment (Sandhu *et al*., 2013).

### 4.2 Combination and maintenance therapies with PARPi for PCa

Published clinical studies have assessed the efficacy of olaparib as a monotherapy in mCRPC only (Fong *et al*., 2009; Kaufman *et al*., 2015; Mateo *et al*., 2015). However, olaparib and possibly other PARPi may be well suited for combination therapies. In particular, combinations with radiotherapy or with DNA‐damaging chemotherapy are considered to add up (Hussain *et al*., 2014; Reiss *et al*., 2015). Here, we were interested whether PARP inhibition combined with first‐line endocrine therapy, that is, ADT and CAB could have superior antitumor effects compared to single treatments. The role of androgenic signaling in generation and repair of dsDNA breaks are currently not fully understood. In line with our results in DuCaP and VCaP (Fig. 3A), activation of AR by dihydrotestosterone modestly decreased sensitivity to olaparib in LNCaP cells (Morra *et al*., 2017). Despite this, it was found that AR reactivation in androgen‐deprived PCa cell lines, including LNCaP and VCaP, induces a transient increase in dsDNA breaks (Hedayati *et al*., 2016). Furthermore, AR is a direct positive regulator of several DNA repair genes and antiandrogens or chemical castration impair error‐prone nonhomologous end joining repair of dsDNA breaks (Polkinghorn *et al*., 2013; Tarish *et al*., 2015). A recent study showed that enzalutamide is also able to decrease expression of selected DNA repair genes involved in HR (Li *et al*., 2017). Here, we could document additive effects of olaparib combined with androgen deprivation on cellular proliferation and apoptosis, albeit only in a cell line possessing numerous aberrations in BRCAness genes, that is, LNCaP (Fig. 4). The effects of AR inactivation on generation of DNA damage/DNA repair activity might therefore be insufficient to generate sensitivity to PARPi. In sum, we conclude that such a combination therapy could be of benefit for PCa patients with known sensitivity to PARPi and receiving first‐line endocrine therapy (ADT or CAB). Thus, similar to olaparib monotherapy, a careful selection of patients in a personalized medicine approach for BRCAness may show best results. Furthermore, an in‐depth analysis of the role of AR in dsDNA break formation and in regulation of DNA repair is warranted in order to be able to conclude on a mechanistic contribution of the AR signaling axis on synthetic lethality with PARPi.

In general, olaparib is well‐tolerated by patients and shows few, mostly grade I‐II adverse effects (Kim *et al*., 2015). Therefore, olaparib and, in general, PARPi are candidates for a maintenance therapy protocol that can be sequenced after an initial tumor‐regressive therapy with the aims to prolong PFS and offering an acceptable quality‐of‐life to patients. Indeed, favorable results in PFS have been noted with olaparib applied as maintenance therapy in ovarian cancer patients with a BRCA mutation after partial or complete response to the most recent platinum treatment (Ledermann *et al*., 2014) without affecting quality‐of‐life (Ledermann *et al*., 2016), compared to placebo‐treated patients. Here, we have addressed the question whether PCa cells are still sensitive to olaparib treatment after initial androgen deprivation. Potential for colony formation, as an indicator for tumor initiation (Fig. 5A), and tumor growth in the RCCS (Fig. 5B) showed that olaparib is still effective after an initial period of androgen removal. We therefore speculate that PARPi could prolong time to castration resistance of mPCa patients on ADT/CAB by sequencing PARPi after initial endocrine therapy. Our data could therefore serve as a basis to design innovative clinical trials to assess olaparib as a maintenance therapy for PCa.

## 5 Conclusions

In summary, olaparib is effective as an anticancer treatment in combination with and as maintenance therapy after first‐line endocrine treatment in PCa cells with markers of BRCAness. ERG rearrangements may not cause and may thus not be suitable as a marker for sensitivity to synthetic lethality with PARPi. Therefore, this preclinical study encourages the design of combination and maintenance therapies with olaparib for endocrine‐treated PCa patients stratified for BRCAness.

## Data accessibility

## Author contributions

GEF, KT, PDL, FG, SP, MH, JB, and FRS acquired and analyzed the data. JV statistically analyzed the data. JB, ZC, and FRS interpreted the data. IS, JB, ZC, and FRS critically revised the manuscript for important intellectual content. FRS performed study design. FRS prepared the manuscript. All authors approved the final version of the manuscript.

## Supporting information


**Fig. S1.** Immunofluorescence for γH2AX foci (red) in DuCaP, VCaP and LNCaP after olaparib treatment. Nuclei were stained with DAPI (blue). White scale bar, 50 μm; red scale bar, 10 μm.

**Data S1.** Supplementary material and methods.

**Table S1.** Medium conditions of the PCa cell lines used in this study and supplementary cell culture conditions.

**Table S2.** Screening for mutations in HR and BRCAness genes using the COSMIC Cell line project′s Mutations database. Determination of BRCA pathogenicity was done with the BRCA mutation and HCI database (University of Utah).

**Table S3.** Screening for expression levels in HR and BRCAness genes using the COSMIC Cell line project′s CNV & Expression database.
