# Supplementary figures and images for: Olaparib is effective in combination with, and as maintenance therapy after, first‐line endocrine therapy in prostate cancer cells
Source: Mol Oncol. 2018 Mar 15;12(4):561–76. doi: 10.1002/1878-0261.12185 (PMC5891051; doi:10.1002/1878-0261.12185)

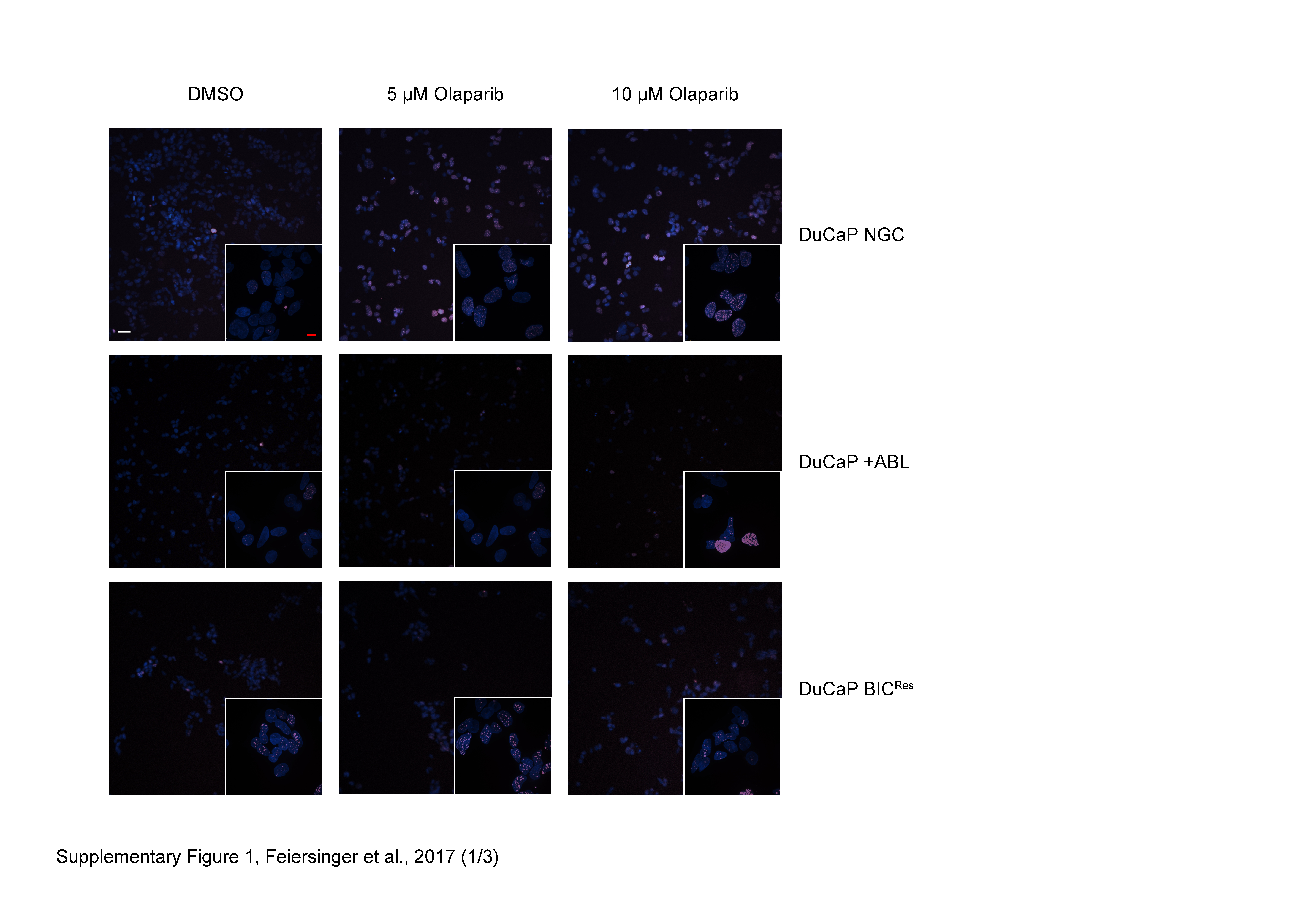

Supplement: Supplementary file 1 — Fig. S1. Immunofluorescence for γH2AX foci (red) in DuCaP, VCaP and LNCaP after olaparib treatment. Nuclei were stained with DAPI (blue). White scale bar, 50 μm; red scale bar, 10 μm. [file MOL2-12-561-s001.jpg]

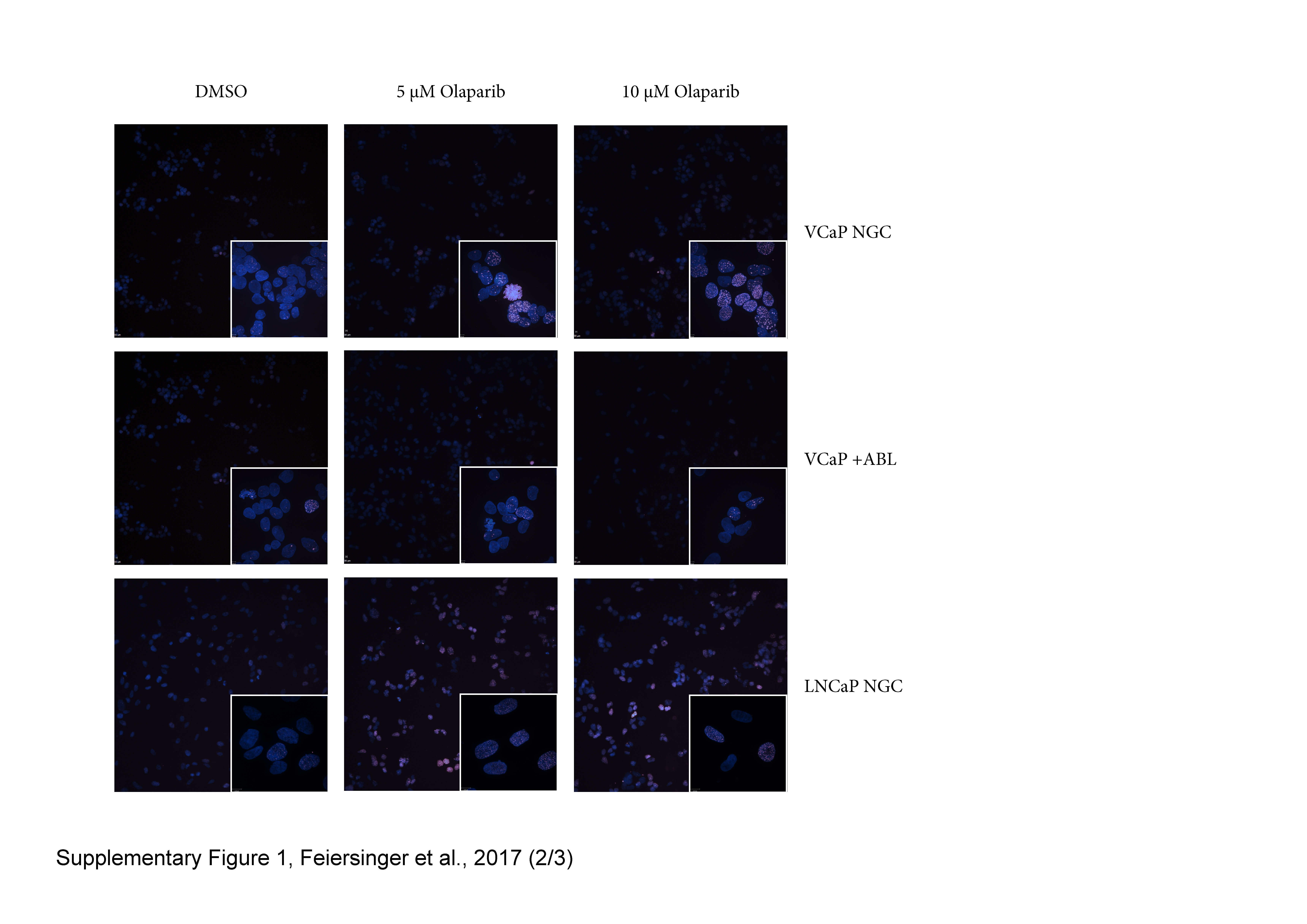

Supplement: Supplementary file 2 [file MOL2-12-561-s002.jpg]

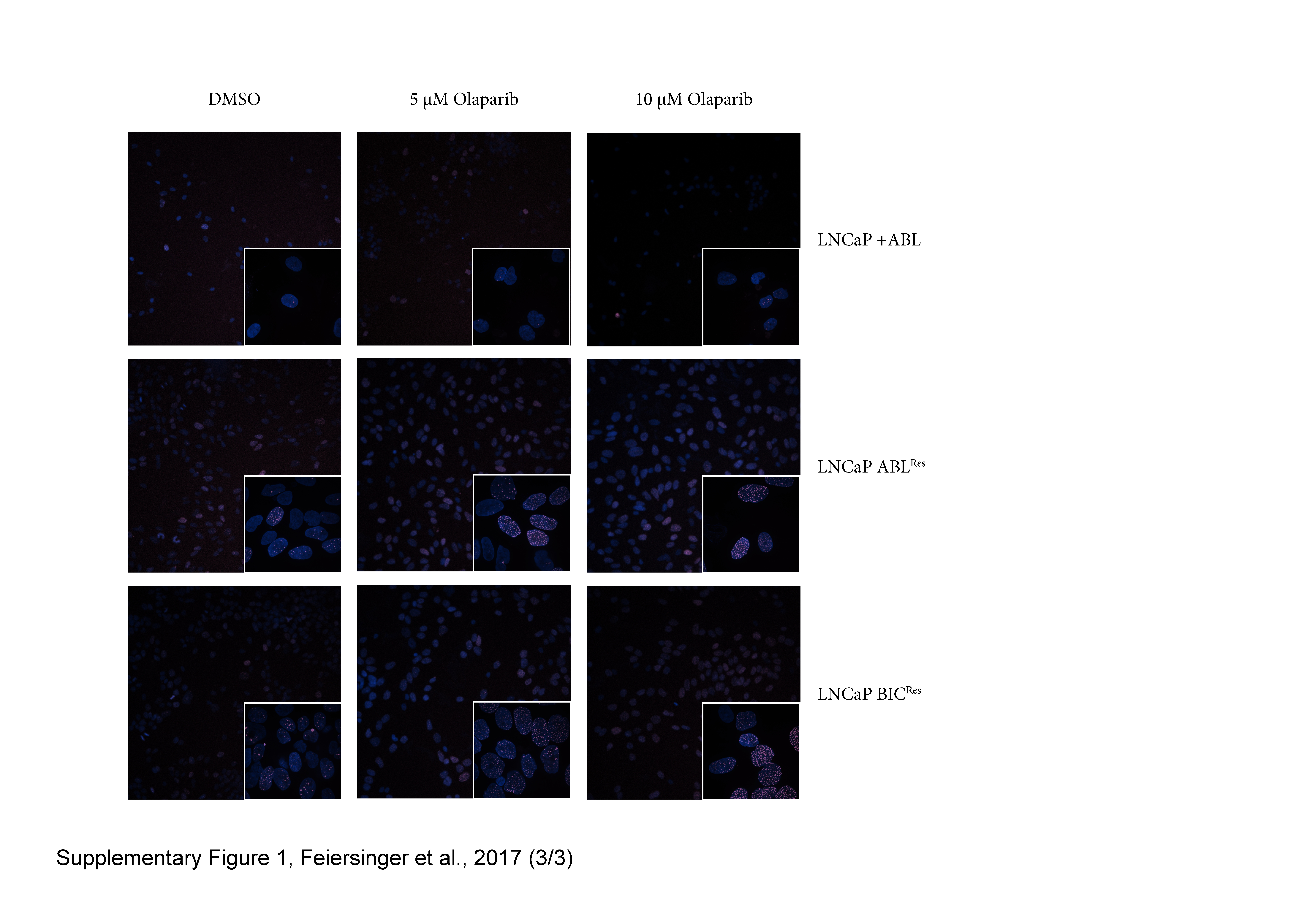

Supplement: Supplementary file 3 [file MOL2-12-561-s003.jpg]
