# Supplementary material for: Olaparib is effective in combination with, and as maintenance therapy after, first‐line endocrine therapy in prostate cancer cells
Source: Mol Oncol. 2018 Mar 15;12(4):561–76. doi: 10.1002/1878-0261.12185 (PMC5891051; doi:10.1002/1878-0261.12185)
Supplement: Supplementary file 4 — Data S1. Supplementary material and methods. [file MOL2-12-561-s004.pdf]

Supplementary Material and Methods

Cell viability and apoptosis assays

Cell lines were seeded into black, flat bottom (apoptosis) or transparent (viability) 96-well plates in duplicates and treated for 96 h with increasing concentrations of Veliparib, Rucaparib, Olaparib or vehicle (DMSO). To measure caspase-dependent induction of apoptosis Caspase 3/7-Glo substrate (Promega) was added to the wells at 1x final concentration. After mixing and 30 min incubation at room temperature light emission was quantified with the luminescence module of a Hidex Chameleon V multiplate reader. Cell viability was assessed by a MTT assay (EZ4U, Biomedica, Vienna, Austria) by measuring the conversion to formazan by living cells. Briefly, cells were incubated for 2 h at 37°C with 10% (v/v) MTT and absorbance of formazan was measured by absorbance at 450 nm using again the Hidex Chameleon multiplate reader. Values are depicted as relative values to DMSO-treated cells in order to allow better comparison between the cell lines.

PSA measurements

Conditioned medium of cells was collected after the indicated incubation times with Olaparib or vehicle. Total PSA in the conditioned medium was determined by the central laboratory of Innsbruck University Hospital by immunoassay on a Cobas 8000 modular analyzer (Roche, Vienna, Austria). Cells were harvested, lysed in RIPA buffer (1 % (v/v) Triton, 0.5 % (w/v) sodium deoxycholat, 0.1 % (w/v) sodium dodecyl sulfate, 150 mM sodium chloride, and 50 mM Tris/Cl pH 8.0) followed by centrifugation at 10,000 g, 5 min, 4°C to pellet cellular debris. Protein concentration in the supernatant was determined by Bradford assay. Total PSA per ml supernatant was normalized on total protein content of lysed cells or to the incubation time of the conditioned medium (Fig. 4A).

^{3}H-thymidine incorporation assays

Cells were seeded in 96-wells at a density of 1.2 x 10^4/well in ≥6 replicates and treated with Olaparib as indicated in the figures. Sixteen hours prior harvesting 1 µCi of ^{3}H-labeled thymidine/well was added. DNA was purified on UniFilter-96GF/C Barex 1.2 µm poresize filter plates (PerkinElmer, Vienna, Austria) using a TomTec 96 mach III harvester (HVD Life Sciences, Vienna, Austria). After addition of 50 µl MicroScint-20 scintillation cocktail (PerkinElmer) light emission was quantified using the liquid scintillation counter module of a Hidex Chameleon V multiplate reader (HVD Life Sciences). Results are expressed in absolute values as relative light units (RLU).

Immunoblotting

Cell extracts were prepared as described under PSA measurements. Twenty-five µg of protein were mixed with LDS-sample loading buffer and reducing agent, heated for 10 min at 70 °C and loaded onto 4-12 % BisTris Gels run at 150 V in 1x MOPS SDS Running Buffer (all Fisher Scientific, Vienna Austria). Proteins were transferred to nitrocellulose membranes by wet-blotting at 40 V for 1 h in 1x Transfer Buffer and blocked with Starting Block (TBS) buffer for 1 h at room temperature (all Fisher Scientific). Membranes were then incubated overnight at 4 °C in Starting Block (TBS) buffer containing 0.1 % (v/v) Tween-20 with the following antibodies: anti-AR N-20 (1:500, sc-816 Santa Cruz, Szabo Scandic, Vienna, Austria), anti-PARP p85 fragment (cPARP, 1:500, G7341 Promega, Mannheim, Germany), anti-ERG [EPR3864] (1:5000, AB92513 Abcam, Cambridge, UK), anti-Glyceraldehyde-3-Phosphate-Dehydrogenase (GAPDH, 1:50,000, MAB374 Millipore, Merck Chemicals, Vienna, Austria) and anti-SV5-Pk1 (V5, 1:2,000, NB100-62264 Novus, Abingdon Oxon, UK). Protein bands were detected by appropriate secondary, fluorescent-labelled antibodies using a near-infrared Odyssey Scanner (Licor, Bad Homburg, Germany).

Confocal microscopy and determination of γH2AX foci number

Cells were seeded in 8-well µ-Slides (Ibidi, Planegg/Martinsried, Germany) at a density of $3x10^5$/well and treated with Olaparib as indicated in the figures. Fixation was performed for 20 min with 4% (w/v) formaldehyde in PBS, followed by a perforation step with 0.2 % (v/v) Triton-X-100 in PBS on ice for 2 min. Cells were blocked with 1% (w/v) bovine serum albumin (AppliChem, VWR, Vienna, Austria) in PBS for 1 h at room temperature (RT) followed by incubation with Alexa Fluor 647-labelled anti-H2AX (pS139) (γH2AX, 1:100, 560447 Becton Dickinson, Schwechat, Austria) for 1 h at RT in blocking buffer. Nuclei were counterstained with Vectashield mounting medium containing DAPI (Vector Laboratories, Szabo Scandic, Vienna, Austria). γH2AX foci were visualized with a spinning disk confocal system (UltraVIEW VoX; Perkin Elmer, Waltham, MA) connected to a Zeiss AxioObserver Z1 microscope (Zeiss, Oberkochen, Germany). Images were acquired with the Volocity software (Perkin Elmer) using a 10x and a 63x oil immersion objective with a numerical aperture of 1.4. Images shown are z-stacks of 10 planes with a spacing of 0.5 µm. Merged images (acquired with the 63x objective) of recorded Z-stacks were analyzed for γH2AX foci number per nucleus using CellProfiler Software (Carpenter et al., 2006) with the Speckle Counting pipeline.

Colony forming assays

Cells were seeded at a density of 3×10^3 per T75 flask and incubated with increasing concentrations of Olaparib for two to three weeks until colonies were clearly visible. Colonies were then fixed with methanol and stained with 0.5 % (w/v) crystal violet in 1:1 methanol:PBS solution. Automated determination of colony number was done with a ColCount camera (Oxford Optronix, Abingdon, UK). Plating efficiency was calculated by the formula: number of colonies / cells seeded x 100.

Lentiviral infection

LNCaP stably overexpressing the truncated form of ERG (as found expressed in canonical TMPRSS2-ERG fusion genes) or V5-lacZ were generated by lentiviral infection using the plasmids pLENTI6.2-ERG (a gift from Prof. A. Chinnaiyan, Ann Arbor, MI, USA) and pLENTI6/V5-GW/lacZ (Fisher Scientific). Viral particles were generated as previously described (Santer et al., 2015). Infected LNCaP cells were selected by 5 µg/ml blasticidin for two weeks and surviving cells were used for subsequent experiments.

References

Carpenter, A.E., Jones, T.R., Lamprecht, M.R., Clarke, C., Kang, I.H., Friman, O., et al., 2006. CellProfiler: image analysis software for identifying and quantifying cell phenotypes. Genome Biology 7R100. doi:10.1186/gb-2006-7-10-r100

Santer, F.R., Erb, H.H., Oh, S.J., Handle, F., Feiersinger, G.E., Luef, B., et al., 2015. Mechanistic rationale for MCL1 inhibition during androgen deprivation therapy. Oncotarget 6(8), 6105–22.
